# Heliotype

**Published:** 1875-06

**Authors:** 


### HELIOTYPE.

This is a sun-picture, as its name indicates. But it differs essentially from the ordinary sun-picture—the photograph. It is a photograph—so to speak—on gelatine, and not on paper, and this gelatine becomes the plate from which printing is done, by the aid of printer's ink and the printing-press.

It reproduces, with all the accuracy of the camera, paintings, engravings, portraits, and views of natural objects; and there is scarcely any form of illustration which may not be rapidly and artistically executed by this remarkable process.

It will reproduce fine steel engravings or woodcuts and all the highest works of art, so accurately and delicately that they can barely be distinguished from the original, and at a price that brings them within the reach of all,—thus making it a great art-educator for the people. It will render an artist's drawing with absolute fidelity, giving his own lines just as he draws them. It will copy faithfully any map, plan, design, or drawing. It will give a perfect fac-simile of any architectural design, whether in lines or tints. It is the only known means of representing faithfully and economically any illustrations of scientific subjects. It will copy statuary and busts with the most beautiful results. It will print lines, light and shade and half-tone, in any colored ink which may be desired. It will faithfully copy any painting, whether of landscape or figure subjects, or any crayon drawing. It will print photographic portraits taken from life, with the most artistic results. It will print photographic views, taken from nature by the camera, so that they can hardly be distinguished from the ordinary photograph. It is especially suitable for all illustrations of botany, natural history, surgery, architecture, engineering, &c. It will accurately copy and represent coins, medals, armor, &c., in the best possible manner, directly from the objects themselves. It is the best and most economical mode of illustrating trade-circulars, catalogues, &c., for jewellers, cabinet-makers, upholsterers, builders, and manufacturers of all classes, and dealers in works of art. From subjects in line, electrotypes can be produced capable of being worked on any printing-press with or without type. Letters and circulars written with ordinary copying-ink on ordinary paper, can be reproduced with absolute fidelity without the intervention of photography.

In all cases, it is to be borne in mind that the reproduction may be fac-simile in size, or enlarged or reduced to any desired extent.

Thus it will be seen that the Heliotype Process (being the application of photography to the printing-press) will accomplish all that photography will accomplish, and much more. It has these additional advantages over photography:—

1. Its impressions are permanent, and will never fade.

2. No mounting is required.

3. The rapidity of production is infinitely greater than that of photography.

4. The cost is very much less than that of photography.

While the foregoing are a few applications of the process, it will be noticed that the Heliotype does not necessarily supplant any existing method of reproduction, but rather supplements and extends the domain of all. The invention is that of Mr. Ernest Edwards, of London, who now superintends the Heliotype Establishment of Messrs. James R. Osgood & Co., of Boston, the well-known publishers, who are the sole owners of the United States Patent. The works are extensive, about fifty presses being kept in constant operation. Here the illustrations of inventions published weekly by the United States Patent-Office are executed.

The American and Foreign Publication Company, the publishers of this journal, are the present agents for this process in the city of New York and vicinity, and have many beautiful specimens of the products of this new and wonderful art on exhibition at their publishing-house, 137 Eighth Street, being aided by Mr. S. E. Temple, of Boston, who is thoroughly familiar with the workings of the process. An examination of these specimens will amply compensate art-students and connoisseurs, as well as all persons who seek the aid of perfect and inexpensive illustrations for business purposes.

---

Domestic Animals.—Persons who have occasion to treat domestic animals in sickness, will find it convenient to know the natural standard of the pulse, as the amount of the deviation from that standard indicates the severity of the disease. In health and repose, the number of pulsations per minute should be as follows: in the horse, 32; ox or cow, 35; sheep, 70; goat, 72; cat, 110; rabbit, 120; dog, 90; duck, 136; hen, 140.

